# 经Ommaya囊脑室内注射培美曲塞治疗非小细胞肺癌软脑膜转移1例及文献回顾

**DOI:** 10.3779/j.issn.1009-3419.2019.08.12

**Published:** 2019-08-20

**Authors:** 永娟 林, 会颖 李, 明敏 黄, 爱斌 郭, 震宇 尹

**Affiliations:** 210008 南京，南京大学医学院附属鼓楼医院老年肿瘤科 Department of Geriatric Oncology, Affiliated Nanjing Drum Tower Hospital of Nanjing University Medical School, Nanjing 210008, China

**Keywords:** 肺肿瘤, 软脑膜转移, Ommaya囊, 培美曲塞, Lung neoplasms, Leptomeningeal metastasis, Ommaya reservoir, Pemetrexed

## Abstract

软脑膜转移（leptomeningeal metastasis, LM）是非小细胞肺癌（non-small cell lung cancer, NSCLC）最严重的并发症之一。随着靶向药物的发展，LM发病率逐年上升，目前缺乏标准有效的治疗方案。鞘内化疗是治疗LM的一种重要方法，但对于NSCLC伴LM，现有的经鞘内途径给化疗药物治疗效果有限，最佳的药物、给药途径、给药模式和剂量仍不清楚。本文报道1例NSCLC伴LM患者，经奥希替尼治疗病情进展后，予培美曲塞经Ommaya囊脑室内化疗，颅内病灶得到较好控制，脑脊液（cerebrospinal fluid, CSF）细胞学转为阴性，同时患者耐受良好，生活质量明显改善，病情长时间内维持稳定，从确诊LM后随访至今已17个月。本文报道了国内外第一例关于经Ommaya囊鞘内注射培美曲塞治疗NSCLC患者LM的临床案例，并结合相关文献总结了鞘内化疗的安全性及有效性，为临床提供了一种LM局部治疗的新策略。

## Abstract (en)

Leptomeningeal metastasis (LM) is one of the most severe complications of non-small-cell lung cancer (NSCLC), and its incidence is increasing gradually with the progress of targeted therapies. There are currently no standard guidelines for the therapy of LM. Intrathecal chemotherapy is the mainstay of treatment for NSCLC patients with LM, but the optimal drug, administration route and mode, and dosage remain unclear. We report a case of LM from NSCLC, who received the intrathecal chemotherapy with pemetrexed by Ommaya reservoir after prior targeted therapies. This local treatment improved the quality of life, and obtained the clearing of CSF cytology and stable lesions of LM without any notable side effects. After confirmation of LM, the patient has survived 17 months until now. Here we report the first case to demonstrate the potential effectiveness of intrathecal pemetrexed by Ommaya reservoir for the treatment of LM of NSCLC, summarize the safety and effectiveness of intrathecal chemotherapy in combination with related literatures, and provide a new strategy for local treatment of LM in clinical.

## 1 病例介绍

患者女性，57岁，无吸烟史，因体检发现右肺占位，2015年8月就诊于南京大学医学院附属鼓楼医院。入院查正电子发射计算机断层显像示右肺上叶后段胸膜下结节代谢增高，考虑肺癌。2015年8月16日行右上肺占位楔形切除术+淋巴结清扫术+胸膜活检术，术后病理示肺腺癌，病理分期IV期（T3N2M1a），表皮生长因子受体（epidermal growth factor receptor, *EGFR*）基因检测示第19外显子缺失。患者于2015年8月24日起口服吉非替尼（易瑞沙，250 mg/d）分子靶向治疗，定期随访复查，病情稳定。2018年1月患者出现一过性癫痫发作、失语，伴有头痛、头晕。头颅磁共振（magnetic resonance imaging, MRI）示左侧额叶局部脑膜增厚（图 1A），进一步行腰椎穿刺术，脑脊液（cerebrospinal fluid, CSF）细胞学检查示大量异形细胞（图 2A），CSF基因测序示*EGFR* 19外显子缺失，考虑肺癌软脑膜转移（leptomeningeal metastasis, LM）。2018年2月起口服奥希替尼（80 mg/d）靶向治疗，之后患者未再发作癫痫，临床症状改善，复查头颅MRI示病灶缩小（图 1B）。2018年8月，患者再次出现头痛，伴有颈项强直，双下肢乏力无法行走。复查CSF仍可见少量异形细胞（图 2B），予加量奥希替尼至160 mg/d，之后患者症状逐渐改善，复查头颅MRI示LM病灶较前缩小（图 1C）。为进一步加强患者LM的控制，2018年11月行Ommaya囊置入术。术后1周拆线，行局部治疗：培美曲塞30 mg，d1、d8，3周方案，同时继续口服奥希替尼（160 mg/d）。具体流程为：局部Ommaya囊充分消毒后插入头皮针，缓慢抽出脑脊液2 mL-5 mL（1 mL/min），予地塞米松5 mg局部预处理，再鞘内缓慢注射培美曲塞30 mg/1 mL，并辅以护胃、止吐、预防癫痫等治疗。经治疗后患者神经系统症状改善明显，可独立行走，复查头颅MRI示病灶基本稳定（图 1D-图 1E），CSF未再查见肿瘤细胞（图 2C）。患者目前仍维持上述治疗，一般情况良好。

**1:**
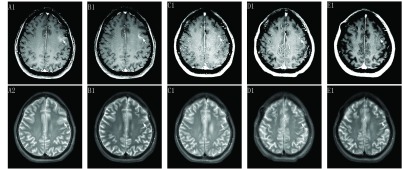
头颅磁共振图像见左侧额叶异常信号，左侧额叶皮层下见短T1稍长T2信号，增强后左侧额叶病灶呈稍高信号。T1w+c增强：A1-E1；T2w相：A2-E2。A：2018年1月首次发现左侧额叶转移灶（白色箭头）；B：2018年5月复查示病灶缩小（白色箭头）；C：2018年10月复查示病灶进一步缩小（白色箭头）；D-E：分别在2019年3月、2019年6月复查示病灶基本维持稳定（白色箭头）。 Craniocerebral MRI scanning revealed abnormalities in the left frontal lobe, short T1 and slightly T2 signal in the left frontal cortex. Enhanced scan revealed that local thickening and obvious enhancement of the meninges in the left frontal lobe. T1w+c: A1-E1; T2w: A2-E2. A: Baseline MRI showed a patch of inhomogeneous enhancement along the sulci (white arrow) in January 2018; B: Repeat MRI in May 2018 showed a reduced lesion (white arrow); C: Repeat MRI in October 2018 showed a reduced lesion (white arrow); D-E: Repeat MRI showed a stable lesion in March and June 2019 respectively (white arrow). MRI: magnetic resonance imaging.

**2:**
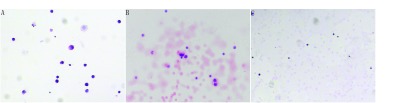
脑脊液涂片（HE染色，×100）。A：2018年1月患者发现脑膜转移后首次腰椎穿刺的脑脊液涂片见较多异形细胞；B：2018年10月复查示少量异形细胞；C：2019年3月复查脑脊液未见异形细胞。 CSF smear (Haematoxylin and eosin staining, 100 times magnification:). A: Baseline CSF smear showed the tumour cells in January 2018; B: Repeat CSF smear found reduced tumour cells in October 2018; C: Repeat CSF smear found no tumour cells in March 2019. CFS: cerebrospinal fluid.

## 2 讨论

LM是非小细胞肺癌（non-small cell lung cancer, NSCLC）最严重的并发症之一，发生率约为5%^[1]^。近年来，由于表皮生长因子受体酪氨酸激酶抑制剂（EGFR-tyrosine kinase inhibitors, EGFR-TKIs）的广泛应用，NSCLC患者的生存期显著延长，LM的发生率也随之逐年增加^[2]^。目前NSCLC相关的LM缺乏标准有效的治疗方案，传统治疗方法如EGFR-TKIs、全身化疗、全脑放疗以及脑室腹腔引流等疗效均不理想，患者预后较差，中位生存期仅3个月左右^[3]^。

目前对于LM伴有*EGFR*驱动基因阳性的NSCLC患者，EGFR-TKIs仍然是首选^[2]^。肺癌伴LM治疗过程中往往需要联合局部治疗（如放疗、鞘内化疗），以进一步提高患者的临床生存获益，局部放疗与鞘内化疗的选择，是综合治疗策略中需要解决的重要问题。由于CSF的循环，无论是局部放疗还是全脑放疗都无法有效清除LM病灶，临床获益甚微^[4]^。局部放疗仅仅是减小局部较大的肿块或者结节，改善压迫症状。血脑屏障可以避免大多数治疗药物进入软脑膜腔，CSF中药物难以达到有效的治疗浓度是LM治疗的最大障碍。鞘内化疗可以直接将抗肿瘤药物送至蛛网膜下腔，对清除肿瘤细胞在软脑膜堆积形成的微小病灶和漂浮在CSF中的肿瘤细胞均更为有效^[5, 6]^，并能减少全身用药带来的严重副反应^[7]^，因而是LM的一种有效治疗手段^[8]^。甲氨蝶呤是最常见的鞘内化疗药物，但针对NSCLC伴LM患者生存获益有限，且化疗毒性大，临床上现已较少使用^[9]^。

培美曲塞是一种新型的多靶点抗叶酸剂，对于非鳞NSCLC具有良好的抗肿瘤活性^[10]^，迄今为止，尚无将培美曲塞用于鞘内化疗的案例。现有研究^[11, 12]^表明，由于培美曲塞的CSF渗透性差，静脉使用时不能有效控制颅内转移病灶。Sun等^[13]^为评估鞘内注射培美曲塞的安全性和药代动力学，在三组小鼠（每组45只）体内留置蛛网膜下腔导管，每组给予不同剂量的培美曲塞（0.3 mg/kg、1 mg/kg和3 mg/kg，每周2次），持续2周，评估鞘内注射培美曲塞的毒副反应。结果证实鞘内注射培美曲塞可在CSF中实现更高、更持久的药物浓度，并且安全可靠。据我们所知，这是国内外第一篇关于脑室内注射培美曲塞治疗NSCLC患者LM的临床报道。患者对该治疗耐受良好，未出现神经毒性相关的症状和体征，且疗效显著，患者神经功能明显改善，生活质量提高，获得较长的无进展生存期。后续期待更多的临床研究进一步优化治疗模式和剂量，证实此治疗方法的安全性及有效性。

鞘内化疗主要的给药途径包括Ommaya囊直接注入脑室及通过腰椎穿刺注入腰椎硬膜囊内。相较于反复腰椎穿刺给药，经Ommaya囊给药存在以下优势：（1）操作更加安全、便捷，患者疼痛少。（2）可依据颅内压行脑脊液引流，及时改善颅高压症状；便于重复送检脑脊液，了解药物浓度及肿瘤指标的动态变化，有助于调整用药、疗效评价及评估预后。（3）药物随脑脊液的循环，可均匀分布于中枢神经系统（central nervous system, CNS）的各个部位，药物浓度可达到同等剂量经腰椎穿刺给药的10倍^[6]^，因而有更好的生存获益^[14]^。基于此，优先推荐选择经Ommaya囊脑室内给药来进行鞘内化疗^[7, 15-17]^。经Ommaya囊鞘内化疗已成功用于治疗多种恶性肿瘤相关的LM（表 1）。Ommaya囊置入术后并发症发生率较低^[18]^，常见的并发症包括：颅内出血、导管易位和堵塞、继发感染以及化疗药物毒副反应等。该患者Ommaya囊置入术后，短期内出现恶心、呕吐以及睡眠障碍，考虑和药物刺激、脑脊液波动相关。

**1:** 经Ommaya囊脑室内注射化疗药物治疗软脑膜转移的研究总结 Summary of studies and cases evaluating intrathecal chemotherapy through Ommaya reservoir for LM

Reference	Primary tumor	*n*	Drug	Complication (%)	Medianbr survival (d)
Bzdil *et al*[22]	Solid tumors	107	NA	9.3	270
Obbens *et al*[23]	Solid tumors	387	NA	6.9	NA
Yoshida *et al*l[24]	Lymphoma >> lung > breast	58	MTX	NA	720
Lavrador *et al*[25]	Breast >> lung > others solid tumor	23	MTX	NA	185
Roguski *et al*[6]	Solid tumor >> Hematologic > primary CNS	80	NA	15.8	72.5
Pardo *et al*[26]	Hematologic	107	MTX liposomal Ara-C	14	NA
Chamberlain *et al*[27]	Breast > lymphoma > melanoma > primary CNS	120	Liposomal cytarabine	11.4	NA
Zairi *et al*[28]	Breast > lung > melanoma	112	Liposomal cytarabine	9.8	NA
Sandberg *et al*[29]	Solid tumors	107	NA	9.3	270
Liaw *et al*[30]	Breast cancer	42	MTX	NA	161
Hitchins *et al*[14]	SCLC > Breast	44	MTX Ara-C	NA	125
Lishner *et al*[31]	Hematologic	106	MTX	8.4	NA
Gwak *et al*[32]	NSCLC	59	MTX	NA	122
CNS: central nervous system; MTX: Methotrexate; cytarabine: Ara-C; SCLC: small cell lung cancer; Median survival (days): Median survival from Ommaya placement to death; Complications: complications related to placement; NSCLC: non-small cell lung cancer. NA：Not available.					

在鞘内化疗的同时，全身性治疗也十分必要，可以有效控制鞘内化疗药物渗透不到的区域及原发病灶。本文患者行鞘内化疗的同时选择继续口服奥希替尼控制颅外病灶。由于其较强的抗肿瘤活性及血脑屏障穿透能力，奥希替尼是*EGFR*敏感突变的NSCLC伴LM转移的一线推荐治疗^[19]^。Bloom研究^[20]^针对NSCLC合并LM推荐奥希替尼剂量为160 mg/d。该患者在口服奥希替尼80 mg/d病情进展后，加量为160 mg/d，原发灶及颅外病灶得到较好的控制。目前NSCLC患者经TKIs治疗后LM的发生机制尚不十分明确，我们无法确定LM的发生是由于获得性耐药，还是由于CSF中奥希替尼仍不能达到有效治疗浓度，使肿瘤细胞找到了“避难所”。研究^[21]^显示，NSCLC患者在TKIs出现获得性耐药后，颅外病灶进展可能性更大。本文患者颅外病灶维持稳定，故我们考虑LM的发生为奥希替尼颅内药物浓度不足导致。这种情况下，继续保留奥希替尼行全身性治疗，同时联合局部化疗，用于治疗晚期NSCLC伴LM是合适的。

综上所述，NSCLC伴LM的发病率逐年上升，患者通常预后较差，尚无有效的治疗手段能有效改善神经功能、延长生存时间。本文报道了首例经Ommaya囊脑室内注射培美曲塞同时联合奥希替尼靶向治疗的病例，患者耐受良好，生活质量明显改善，疗效肯定。此治疗方案的有效性及安全性、最佳的治疗模式及药物剂量，需在*EGFR*敏感突变的NSCLC伴LM患者中进一步探索、证实。
